# Combinations of Physiologic Estrogens with Xenoestrogens Alter ERK Phosphorylation Profiles in Rat Pituitary Cells

**DOI:** 10.1289/ehp.1002512

**Published:** 2010-09-22

**Authors:** Yow-Jiun Jeng, Cheryl S. Watson

**Affiliations:** Department of Biochemistry and Molecular Biology, University of Texas Medical Branch, Galveston, Texas, USA

**Keywords:** ERα, ERβ, ERK activation, GPER, membrane estrogen receptors, nongenomic effects, physiologic estrogens, prolactinoma cell line, xenoestrogens

## Abstract

**Background:**

Estrogens are potent nongenomic phospho-activators of extracellular-signal–regulated kinases (ERKs). A major concern about the toxicity of xenoestrogens (XEs) is potential alteration of responses to physiologic estrogens when XEs are present simultaneously.

**Objectives:**

We examined estrogen-induced ERK activation, comparing the abilities of structurally related XEs (alkylphenols and bisphenol A) to alter ERK responses induced by physiologic concentrations (1 nM) of estradiol (E_2_), estrone (E_1_), and estriol (E_3_).

**Methods:**

We quantified hormone/mimetic-induced ERK phosphorylations in the GH_3_/B6/F10 rat pituitary cell line using a plate immunoassay, comparing effects with those on cell proliferation and by estrogen receptor subtype-selective ligands.

**Results:**

Alone, these structurally related XEs activate ERKs in an oscillating temporal pattern similar (but not identical) to that with physiologic estrogens. The potency of all estrogens was similar (active between femtomolar and nanomolar concentrations). XEs potently disrupted physiologic estrogen signaling at low, environmentally relevant concentrations. Generally, XEs potentiated (at the lowest, subpicomolar concentrations) and attenuated (at the highest, picomolar to 100 nM concentrations) the actions of the physiologic estrogens. Some XEs showed pronounced nonmonotonic responses/inhibitions. The phosphorylated ERK and proliferative responses to receptor-selective ligands were only partially correlated.

**Conclusions:**

XEs are both imperfect potent estrogens and endocrine disruptors; the more efficacious an XE, the more it disrupts actions of physiologic estrogens. This ability to disrupt physiologic estrogen signaling suggests that XEs may disturb normal functioning at life stages where actions of particular estrogens are important (e.g., development, reproductive cycling, pregnancy, menopause).

The physiologic estrogens estradiol (E_2_), estrone (E_1_), and estriol (E_3_) play important and selective roles in different life stages of women. E_2_ is the predominant form driving sexual development and function of reproductive organs (e.g., breast and uterus) and the menstrual cycle. E_1_ and E_3_ have important physiologic functions at discrete life stages such as pregnancy, and E_1_ is a major estrogen in the postmenopausal phase. These estrogens are produced mainly in the ovaries and in the placenta, although small amounts are also produced by the adrenal glands and brain (Lippert et al. 2003; Reddy 1979). Some E_1_ is made in most tissues, but especially in fat and muscle, which are a major source of estrogens in postmenopausal women. All of these physiologic estrogens may also affect the brain, bone, cardiovascular system, and many other tissues (Cornwell et al. 2004). Although important for physiologic control of female development and reproduction, and the coordination of many other tissues and organ systems to support reproduction, estrogens are also associated with the development of cancers of estrogen-responsive tissues such as breast, uterus, and pituitary. Therefore, it is important to understand mechanisms that affect the balance between these beneficial versus detrimental roles of estrogens.

Mitogen-activated protein kinases (MAPKs), including extracellular-signal–regulated kinases (ERKs), play central roles in controlling such diverse cellular outcomes as cell proliferation, apoptosis, and maturation to perform distinct functional roles. Such kinases can also influence whether cells proliferate excessively and develop into tumors. Many different stimuli, including steroids, growth factors, cytokines, ligands for G-protein–coupled receptors, and carcinogens, can activate the ERK pathways; ERKs can therefore serve as signal integrators for all of these inputs (Watson et al. 2010), so that the cell can render a final decision about its overall destiny (cell division, differentiation, death, or malignant transformation). The contribution of physiologic estrogens to this signaling mixture, integrated by MAPKs, can also be influenced by estrogens that signal inappropriately, such as xenoestrogens (XEs).

In a rat pituitary cancer cell subline (GH_3_/B6/F10) that we selected for its naturally high levels of membrane estrogen receptor-α (mERα) expression (Pappas et al. 1994), treatment with E_2_ can activate ERKs and also cause prolactin release within a few minutes, elicited in part by calcium influx within seconds (Bulayeva et al. 2004, 2005; Norfleet et al. 2000; Pappas et al. 1995a). Rapid inductions of ERK phosphorylation by E_2_ are contributed to by many different upstream signaling pathways, with signals traveling down their cascades at varying speeds (Belcheva and Coscia 2002; Bulayeva et al. 2004). Although the less studied physiologic estrogens E_1_ and E_3_ have weak effects on transcription (Kuiper et al. 1997; Lippman et al. 1977), they are as effective as E_2_ in causing ERK phosphorylation and other nongenomic responses in pituitary, breast, uterine, and neuronal cells (Alyea and Watson 2009b; Kabil et al. 2008; Mermelstein et al. 1996; Raz et al. 2008; Watson et al. 2008; Zhang et al. 2009). In the present study we address the interaction of physiologic estrogens and alkylphenol-class XEs on ERK activation.

Environmental estrogens are manufactured products that contaminate food, water, or air to which humans and animals are exposed (Foster 2008; Sonnenschein and Soto 1998; Tapiero et al. 2002). The alkylphenols are a structurally related (and therefore interesting to compare mechanistically) set of such compounds; they vary in their carbon side-chain length or, in the case of bisphenol A (BPA), have a phenol group instead of an alkyl side chain [see Supplemental Material (doi:10.1289/ehp.1002512)]. Alkylphenols are widespread industrial surfactants (Burback and Perry 1993; Kolpin et al. 2002; White et al. 1994), whereas BPA is ubiquitous because it is a precursor and breakdown product of the commonly used polycarbonate plastics (Tsai 2006). Many environmental estrogens readily accumulate in the environment and in animal tissues and have been shown to affect both reproductive and nonreproductive organs/systems in animals and humans (Han et al. 2004; Hossaini et al. 2001; Moon et al. 2007; Tsai 2006). Although the genomic effects of these environmental estrogens have been studied extensively and found to be weak (causing government regulatory agencies to consider them “safe”), until recently little was known about their nongenomic estrogenic effects. Environmental estrogens produce potent membrane-initiated signaling effects similar but not identical to those elicited by E_2_ (Alyea and Watson 2009a; Bulayeva and Watson 2004; Kabil et al. 2008; Wozniak et al. 2005; Zsarnovszky et al. 2005). A prominent characteristic that has greatly contributed to the confusion over their toxicity is the nonmonotonic concentration dependence of their nongenomic responses (Palanza et al. 2001; Vandenberg et al. 2006; Watson et al. 2010). Environmental estrogens are suspected of affecting a wide variety of functions by interfering with the actions of physiologic estrogens, but sufficiently sensitive techniques for quantitative documentation of these disruptions have generally been lacking.

In the present study, we examined whether diverse physiologic estrogens and XEs [whose structures are shown in Supplemental Material (doi:10.1289/ehp.1002512)] can disrupt physiologic estrogen signaling via ERKs when in combination. We studied all compounds at a wide range of low concentrations and at multiple time points because we expected variations in both their concentration dependence and response progression. We then examined which estrogen receptor (ER) subtypes participate in these ERK and proliferation responses. We used fixed cell-based immunoassays that allowed us to analyze many conditions simultaneously. By examining these mER-mediated cellular responses leading to the integrated activation of ERKs, we hoped to better understand the endocrine-disrupting impact of the alkylphenol class of environmental XEs.

## Materials and Methods

### Materials and cell culture

We purchased phenol red-free Dulbecco’s modified Eagle medium (DMEM; high glucose) from Mediatech (Herndon, VA), horse serum from Gibco BRL (Grand Island, NY), and defined supplemented calf and fetal bovine sera from Hyclone (Logan, UT). Paraformaldehyde and picric acid were purchased from Fisher Scientific (Pittsburgh, PA). The antibody used to measure phosphorylated ERKs 1 and 2 was purchased from Cell Signaling Technology (Danvers, MA), and the ERα-selective agonist 4,4′,4″-[4-propyl-[1H]-pyrazole-1,3,5-triyl] trisphenol (PPT) and the ERβ-selective agonist 2,3-bis[4-hydroxyphenyl]-propionitrile (DPN) were purchased from Tocris (Ballwin, MO). The G-protein–coupled ER (GPER) agonist G1 was a gift from Chemdiv Inc. (San Diego, CA) arranged by E. Prossnitz. Pertussis toxin, nystatin, BPA, 4-*n*-ethylphenol (EP), 4-*n*-propylphenol (PP), 4-*n*-octylphenol (OP), 4-*n*-nonylphenol (NP), and other chemicals were purchased from Sigma (St. Louis, MO). GH_3_/B6/F10 cells were routinely propagated in DMEM containing 12.5% horse serum, 2.5% defined supplemented calf serum, and 1.5% fetal calf serum. Cells were used between passages 10 and 20.

### Quantitative ERK phosphorylation assays

We developed this assay to assess levels of activated ERKs 1 and 2 in fixed GH_3_/B6/F10 cells (Bulayeva et al. 2004). Briefly, cells were plated at 10,000 cells/poly-d-lysine–coated well in 96-well plates. The next day growth medium was replaced with DMEM containing 1% charcoal-stripped (4×) serum for 48 hr. Washed cells were then treated with different estrogenic compounds for 5 min. For receptor subtype identification, we treated cells with ERα versus ERβ versus GPER analogs for 5 min before assay of phosphorylated ERK (pERK). We routinely used 20 nM phorbol 12-myristate 13-acetate (TPA) as a positive control to demonstrate maximum activation of ERKs via the protein kinase C pathway; for comparison, activation by E_2_ generally achieved 85% of the activation levels by TPA. The cells were then fixed with 2% paraformaldehyde/0.2% picric acid at 4°C for 48 hr and then permeabilized with phosphate-buffered saline (PBS) containing 2% bovine serum albumin (BSA)/0.1% Triton X-100 for 1 hr at room temperature. Cells were then washed three times with PBS and treated with primary antibody against pERKs (p-Thr202/Tyr204; 1:400 in PBS/1% BSA). After overnight incubation at 4°C, the cells were processed for signal development with the Vectastain ABC kit (Vector Laboratories, Burlingame, CA) following manufacturer recommendations. Biotin-conjugated secondary antibody was used at a 1:300 dilution. Plates were incubated in the dark for 20 min at 37°C for the generation of alkaline phosphatase product (*para*-nitrophenol) and then read at A_405_ in a model 1420 Wallace microplate reader (Perkin Elmer, Waltham, MA). We used the crystal violet assay to estimate the number of cells in each well for signal normalization. Experiments were repeated at least two to three times using different passages of cells on different days.

### Crystal violet assay

After ERK assays, the quantitative immunoassay reagents were washed from the multiwell plates and the cells were stained with 0.1% crystal violet for 30 min, destained with deionized water, and extracted (10% acetic acid) following the method of Lottering et al. (1992). The A_590_ signal of the extract was read in a model 1420 Wallace microplate reader. This assay was also used for proliferation studies to approximate cell number.

### Statistics

Data from pERK studies were analyzed by one-way analysis of variance followed by multiple comparisons with the control group (Holm–Sidak method) using Sigma Stat (version 3; Systat Software Inc., Chicago, IL). Significance was accepted at *p* < 0.05.

## Results

**Figure 1:**
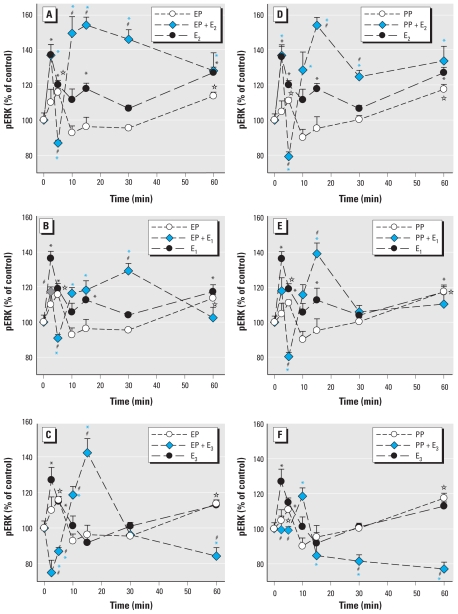
Time-dependent changes in pERK elicited by combinations of short-chain alkylphenols with E_2_, E_1_, or E_3_. GH_3_/B6/F10 cells were cotreated with 1 nM EP (*A*–*C*) or PP (*D–F*) and 1 nM E_2_ (*A*,*D*), E_1_ (*B*,*E*), or E_3_ (*C*,*F*). The pERK levels were measured by plate immunoassay after different times of cotreatment. *p < 0.05 compared with vehicle-treated control cells, shaded to match each set of data symbols (*, 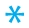, ✫). ^#^*p* < 0.05 compared with cells treated with 1 nM E_2_, E_1_, or E_3_ alone.

**Figure 2:**
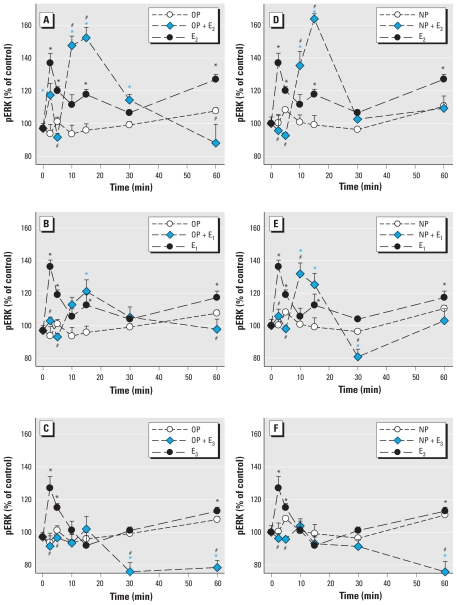
Time-dependent changes in pERK elicited by combinations of long-chain alkylphenols with E_2_, E_1_, or E_3_. GH_3_/B6/F10 cells were cotreated with 1 nM OP (*A*–*C*) or NP (*D*–*F*) and 1 nM E_2_ (*A*,*D*), E_1_ (*B*,*E*), or E_3_ (*C*,*F*). The pERK levels were measured by plate immunoassay after different times of cotreatment. *p < 0.05 compared with vehicle-treated control cells, shaded to match each set of data symbols (*, 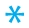). ^#^*p* < 0.05 compared with cells treated with 1 nM E_2_, E_1_, or E_3_ alone.

**Figure 3:**
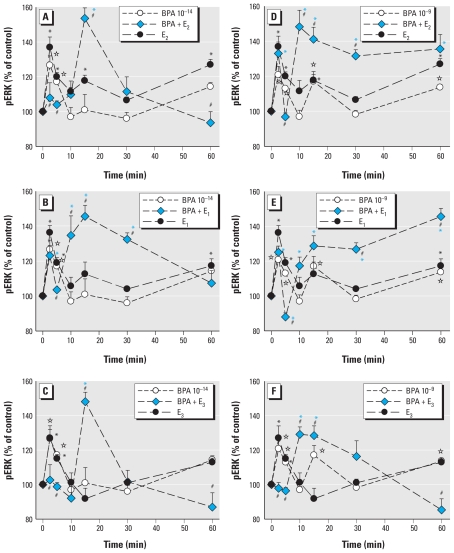
Time-dependent changes in pERK elicited by combinations of BPA at two different environmentally relevant concentrations. GH_3_/B6/F10 cells were cotreated with 10^−14^ M BPA (*A*–*C*) or 10^−9^ M BPA (*D*–*F*) plus 1 nM E_2_ (*A,D*), E_1_ (*B*,*E*), or E_3_ (*C*,*F*). The pERK levels were measured after different times of cotreatment. *p < 0.05 compared with vehicle-treated control cells, shaded to match each set of data symbols (*, 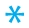, ✫). #*p* < 0.05 compared with cells treated with 1 nM E_2_, E_1_, or E_3_ alone.

Because women are exposed to environmental estrogens during life phases when different physiologic estrogens are prevalent, we studied the changes in pERK when each physiologic and environmental estrogen was present simultaneously. We previously detailed ERK (and other) responses of these cells to the physiologic estrogens E_1_, E_2_, and E_3_ (Watson et al. 2008); those data can be directly compared with results of the present study. First, we examined the time-dependent changes in ERKs (Figures 1–3), showing that estrogenic stimulation caused a characteristic oscillating pattern with immediate (5 min), intermediate (10–30 min), and long-term (after 30 min) rises in ERK activation, similar to estrogen-induced fluctuations we reported previously (Bulayeva et al. 2004; Bulayeva and Watson 2004; Jeng et al. 2009; Jeng and Watson 2009; Kochukov et al. 2009; Zivadinovic and Watson 2005). We observed these oscillating patterns for all estrogens, although some were “trends” with peaks that did not achieve significance. The physiologic estrogens E_2_ and E_1_, as well as BPA, tended to cause three oscillations during this 60-min time frame, whereas alkylphenols and E_3_ caused only two (missing the intermediate peak).

When pituitary cells were cotreated with XEs plus each of the physiologic estrogens (E_2_, E_1_, or E_3_) in combination, the usual 2.5- to 5-min pERK peak caused by individual estrogens was often abolished or blunted. Instead, the combination of compounds usually caused a pronounced early dephosphorylation and then created a new time-delayed, augmented phosphorylation peak, just as the actions of individual estrogens waned. These new large activations (although a bit smaller in the PP, OP, and NP combinations with E_3_) peaked instead in the intermediate 10- to 30-min time frame, demonstrating how combinations of estrogens with XEs that individually do not elicit a significant response at a given time point can cause a synergistic response. Then, as the ERK activation induced by individual estrogens again rose at 60 min, the level due to the combined estrogens usually declined, often far below the response to the individual estrogens. (The exceptions to this observation were combinations of PP plus E_2_ and 10^−9^ M BPA plus E1 or E_2_, when the intermediate pERK peak was sustained.) Although the later (30–60 min) response period was usually inhibited, it depended somewhat on the efficacy of the XE at that time point. That is, stronger individual XE responses (resembling physiologic estrogen responses) tended to predict the ability of the XE to inhibit the actions of physiologic estrogens when in combination. Thus, a three-peaked oscillation caused by physiologic estrogens was transformed to a single major intermediate peak of activation in most cases.

We used two doses of BPA to determine the effects on ERK activation caused by physiologic estrogens (Figure 3) in our temporal phase studies because BPA is a compound of high interest to the endocrine toxicology community due to its ubiquity and the controversy about its potential effects, and because both of the chosen concentrations are in the range of typical and prevalent exposures. More important, many estrogens, especially BPA, cause nonmonotonic nongenomic dose–response patterns, typically having multiple dose optima. Interestingly, the very low concentration of BPA (10^−14^ M) caused a two-phased oscillating response rather like the alkylphenol XEs (Figures 1 and 2). However, 1 nM BPA elicited a response more typical of the physiologic estrogen pattern (with three phases of activation).

Although we could choose a single health-relevant level of physiologic estrogens based on their normal concentration ranges in animals and humans (Greenspan and Gardner 2004), we felt it necessary to test many concentrations of the XEs to adequately describe the effects they might have at different common contamination levels, and to account for their nonmonotonic behavior. We chose the 5-min time point for this study because of its prominent and consistent appearance in all physiologic estrogen and XE treatments (Figures 1–3) and because its rapid time frame ensured that it would result in a distinctly nongenomic response.

**Figure 4:**
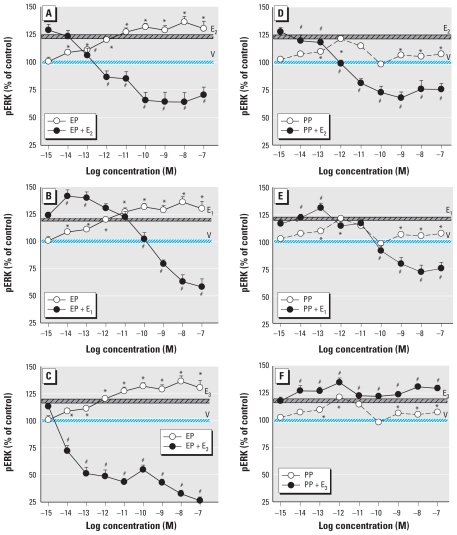
Concentration-dependent changes in pERK caused by short-chain alkylphenols. Cells were treated for 5 min with a combination of 1 nM E_2_ (*A*,*D*), E_1_ (*B*,*E*), or E_3_ (*C*,*F*) plus different concentrations of EP (*A*–*C*) or PP (*D*–*F*), and pERK was assayed. The blue horizontal bar indicates the pERK level and error range in vehicle-treated cells (V); the crosshatched horizontal bar indicates the pERK value in cells treated with nanomolar concentrations of E_2_, E_1_, or E_3_ alone. *p < 0.05 compared with vehicle-treated cells. ^#^*p* < 0.05 compared with cells treated with E_2_, E_1_, or E_3_ alone.

**Figure 5:**
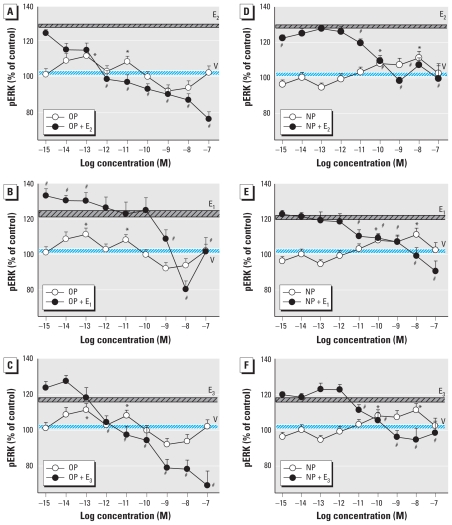
Concentration-dependent changes in pERK caused by the long-chain alkylphenols. Cells were treated for 5 min with a combination of 1 nM E_2_ (*A*,*D*), E_1_ (*B*,*E*), or E_3_ (*C*,*F*) plus different concentrations of OP (*A*–*D*) or NP (*D*–*F*), and pERK was assayed. The blue horizontal bar indicates the pERK level and error range in vehicle-treated cells (V); the crosshatched horizontal bar indicates the pERK value in cells treated with nanomolar concentrations of E_2_, E_1_, or E_3_ alone. *p < 0.05 compared with vehicle-treated cells. #*p* < 0.05 compared with cells treated with E_2_, E_1_, or E_3_ alone.

For most alkylphenols acting alone (EP, PP, and NP; Figures 4 and 5), significant estrogenic effects occurred at the higher doses (usually picomolar or higher concentrations), but OP showed more activity at the lower doses. The short-chain EP caused greater stimulations than did all physiologic estrogens. PP caused marked nonmonotonic dose responses, with effective doses peaking in both approximately picomolar and nanomolar ranges. Alkylphenols generally enhanced physiologic estrogen responses at lower doses but severely disrupted them at higher doses to far below vehicle control levels. EP was estrogenic over most of the tested concentration range and inhibited its paired physiologic estrogens throughout. PP was significantly estrogenic at two concentration ranges and lowered the estrogenicity of E_2_ and E_1_ beginning at the concentration at which it became estrogenic itself (approximately picomolar). However, PP enhanced the estrogenicity of E_3_ at all but the lowest tested concentrations (Figure 4F). This last result was the most significant departure from the general pattern and illustrates why each compound must be tested across its entire range to reveal such exceptions.

**Figure 6:**
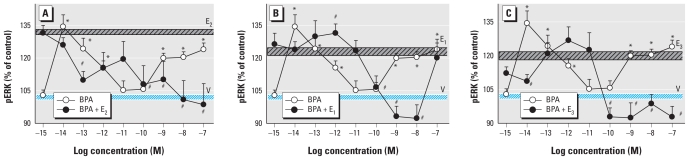
Concentration-dependent alteration of physiologic estrogen-induced pERK by BPA. Cells were treated for 5 min with different concentrations of BPA with or without 1 nM E_2_ (*A*), E_1_ (*B*), or E_3_ (*C*). The blue horizontal bar indicates the pERK level and error range in vehicle-treated cells (V); the crosshatched horizontal bar indicates the pERK value in cells treated with nanomolar concentrations of E_2_, E_1_, or E_3_ alone. *p < 0.05 compared with vehicle-treated cells. ^#^*p* < 0.05 compared with cells treated with E_2_, E_1_, or E_3_ alone.

BPA showed the most striking nonmonotonicity in its dose–response pattern (Figure 6), as we have seen previously in this and other cell systems (Alyea and Watson 2009a; Watson et al. 1999). The lowest BPA doses were very estrogenic, followed by a (generally) 2 log dose range of ineffective concentrations (10–100 pM), followed finally by another dose range of estrogenic activity (at nanomolar or higher concentrations). Remarkably, at whatever doses BPA was most estrogenic, it had the most marked inhibitory effect on the estrogenicity of the paired physiologic estrogen. At doses where BPA was ineffective, the estrogenicity of the physiologic estrogen was spared (close to normal or, for E_2_, at least clearly above vehicle control values) or even enhanced (for E_1_ and E_3_).

**Figure 7:**
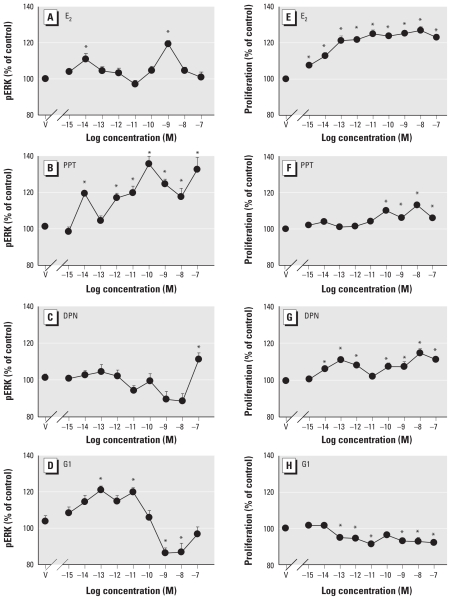
Concentration dependence of pERK (*A–D*) and proliferation (*E–H*) on selective ER agonists. pERK was measured in cells after 5 min of treatment with different concentrations of E_2_ (*A*), the ERα agonist PPT (*B*), the ERβ agonist DPN (*C*), or the GPER agonist G1 (*D*). Proliferation was measured (via the crystal violet assay) in cells treated for 3 days with matching concentrations of E_2_ (*E*), PPT (*F*), DPN (*G*), or G1 (*H*). V, vehicle-treated cells. *p < 0.05 compared with vehicle-treated cells.

To further characterize this response caused by all estrogens, we examined in more detail which of several ER proteins were responsible (Figure 7A–D). We again examined ERK activation after 5 min, as a distinct nongenomic response indicator. The selective ERα agonist PPT increased pERK levels over a wide range of concentrations (as low as 10^−14^ M), including those highly selective for ERα. The ERβ agonist DPN had no effect at any selective concentration; it is known to be rather nonselective at higher levels (Meyers et al. 2001). The selective GPER agonist G1 increased pERK levels at relatively low concentrations (approximately picomolar) but decreased pERK at higher than nanomolar concentrations.

Because ERK activation is often associated with the proliferative response, we also used these same receptor-selective compounds to evaluate their ability to evoke cell proliferation after 3 days of treatment (Figure 7E–H). At selective concentrations, both PPT and DPN caused cell proliferation, whereas G1 caused a decrease in cell numbers at all concentrations ≥ 100 fM. In our side-by-side comparison, ERK activation correlated with the ability to affect proliferation reasonably well for PPT and negatively for G1 but did not correlate well with cell proliferation caused by DPN (which also did not selectively activate ERK). Because E_2_ activates all forms of ERs, it has a composite (positive) profile for inducing cell proliferation.

## Discussion

Oscillating time-dependent and nonmonotonic concentration-dependent responses are typical for nongenomic estrogenic responses (Alyea et al. 2008; Watson et al. 2007a, 2010; Zsarnovszky et al. 2005), and we again demonstrated such characteristics for this set of structurally related XEs of the alkylphenol class. The complexities of fluctuating responses and their relationship to multiple signaling pathway involvement in the activation of ERKs by estrogens have only recently been appreciated. Such complications require that we develop methods to document different functional and signaling outcomes quantitatively, in order to establish the different potencies and temporal patterns evoked by XEs, as we did in this study. Our quantitative ERK activation assays allowed multiple comparisons between structurally related physiologic estrogens (E_1_, E_2_, E_3_) and an XE class (alkylphenols and BPA), and also allowed us to carefully document the combinatorial effects of XEs (both inhibiting and enhancing estrogenicity) on these physiologic estrogens that are functionally important to different life stages of women and men.

By themselves, all three physiologic estrogens, BPA, and alkylphenols were potent nongenomic response activators at the low concentrations tested, as we have seen previously (Alyea and Watson 2009a; Bulayeva and Watson 2004; Kochukov et al. 2009; Midoro-Horiuti et al. 2010; Narita et al. 2007; Watson et al. 2008; Wozniak et al. 2005). In contrast, many past studies of genomic responses to these compounds (other than E_2_) showed them to be weak (Bonefeld-Jørgensen et al. 2007; Moon et al. 2007). Signaling or functional response profiles for different nongenomic responses can vary markedly among estrogens (Borgert et al. 2003; Kochukov et al. 2009; Routledge et al. 2000), as we demonstrated again in this study, so each must be examined individually until such time as our structure–activity knowledge increases.

XEs have been shown to disrupt physiologic estrogenic responses *in vivo* (Alonso-Magdalena et al. 2006; Midoro-Horiuti et al. 2010). Testing of low, environmentally relevant concentrations was prompted in many cases by the demonstration that they are active on cell signaling responses and functions *in vitro* (Alyea and Watson 2009a; Bulayeva and Watson 2004; Kochukov et al. 2009; Watson et al. 2007b; Wetherill et al. 2007; Wozniak et al. 2005). However, little is currently known about exactly how such complex signaling patterns are initiated by XEs. Their weak affinity profiles for the nuclear versions of ERs (Blair et al. 2000) are puzzling, given their potent actions via membrane versions of the same receptors. Binding of such lipophilic compounds to membrane-resident proteins is difficult to measure and interpret because of high backgrounds in the measurements and small populations of membrane-resident receptors (Nadal et al. 2000; Powell et al. 1999), as well as possible binding to different-shaped binding pockets.

The estrogenicity of all physiologic estrogens examined in our assay was subject to similar disruptions by combination with XEs. Levels of pERK were either attenuated or potentiated, depending on the timing or concentration of the treatments, making clear the necessity of examining these parameters carefully for each compound. However, generally speaking, some overall principles were established. Whenever an XE caused a strong response (either at a particular time point or concentration), the paired physiologic estrogen response was inhibited; whenever an XE caused a weak or no response, its combination with physiologic estrogens usually caused a synergistic enhancement of pERK levels. This was particularly easy to observe for BPA, with its pronounced nonmonotonic dose curve, but we also observed it in the responses to the other alkylphenols. Cotreatments often resulted in out-of-phase oscillations compared with those caused by individual estrogens, with apparent shifts to later response times and less frequent oscillations. In another (neuronal) cell type exhibiting a different functional response (dopamine efflux), we saw a similar pattern, especially with low concentrations of XEs in combination with E_2_ (Alyea and Watson 2009a).

We used very low doses of BPA in the present study compared with many others in the literature; this emphasizes the toxicity that can be caused by levels commonly present in our environment and food. BPA potentiated ERK activation at levels as low as picomolar concentrations, whereas femtomolar and nanomolar BPA generally disrupted all physiologic estrogen-induced ERK activities. Changes seen in later (60 min) ERK activation with several estrogen/XE combinations may indicate functional consequences for regulating cell proliferation (Razidlo et al. 2004; Yang et al. 2008) and other epigenetic aspects of carcinogenicity (Bredfeldt et al. 2010). Even though we tested some responses only at short exposure times to emphasize the rapid nature of these activations and disruptions, such stimuli would undoubtedly be sustained or repeated, because these compounds generally persist both in fat stores and in the environment (Calafat et al. 2005; Ye et al. 2006).

Using selective ER agonists, we found that ERα activation was the dominant mediator of the pERK level increases, whereas ERβ had no effect. The activation by the selective GPER agonist G1 is difficult to interpret because it caused a biphasic activation of ERK, being stimulatory only at very low concentrations (femtomolar to picomolar) but inactivating ERK at picomolar to nanomolar ranges. Although natural ligands (other than the nonselective E_2_) are not known for GPER, these data suggest an inhibitory role for GPER at what would be an expected physiologic concentration of hormone acting on adult tissue (up to nanomolar concentrations). GPER’s sensitive activation by G1 does not match what is known about its affinity for G1 and its activation of other functional end points (Bologa et al. 2006; Filardo et al. 2000; Prossnitz and Maggiolini 2009; Thomas et al. 2005). Could this indicate an important role for GPER in other, less-studied scenarios such as developmental signaling, where hormonal levels generally rise from nonexistent to initially quite low levels to trigger tissue remodeling? Do cultured cancer cells exhibit receptor subtypes and sensitive responses that are generally more common to developmentally immature cells and so have an exaggerated GPER response? Finally, there are now reports that G1 is actually a ligand for an alternatively spliced/truncated form of ERα (ERα36) that interacts with GPER, but not for GPER itself (Kang et al. 2010), and that this form is involved in the activation of ERKs. Interestingly, our data show activation via both ERα and GPER (but not ERβ) and do not exclude the interpretation that G1 acts via ERα36 in our cell line. Antibodies that we have used in the past to identify mERα on these cells and to select high expressers [e.g., H222; (Pappas et al. 1995a, 1995b)] also recognize ERα36, and our immunoblots of membrane proteins with H222 show multiple immunoreactive proteins, including one that is 36 kDa (data not shown). Thus, our cell line probably also expresses this form.

Estrogens and XEs cause these cells to proliferate and also activate ERKs and other MAPKs, as we have shown previously (Jeng and Watson 2009; Kochukov et al. 2009; Watson et al. 2008), although some phytoestrogens inhibit proliferation when combined with physiologic estrogens (Jeng et al. 2010; Jeng et al. 2009). Often ERKs are designated as the MAPKs most responsible for cell proliferation, with some exceptions to this generalization (Zhou et al. 2007). In the present study the ERα-selective ligand PPT caused both ERK phosphorylation and proliferation, consistent with that hypothesis, whereas selective GPER liganding mostly inhibited both processes at expected physiologic concentrations. Although the ERβ ligand did not evoke an ERK response at selective concentrations, it was still capable of causing some cell proliferation at a wide range of concentrations, some selective. Clearly, these considerations are more complicated than we usually summarize them to be. The balance of different kinds of MAPKs activated by these compounds could tell a more complete story, because sometimes the ability of estrogens to activate ERKs 1 and 2 compared with other ERKS, c-Jun N-terminal kinases (JNKs) and p38 differ.

Phosphorylation of cellular ERKs is a dynamic process, involving multiple pathways and regulators in complicated time- and concentration-dependent patterns, creating oscillating ERK (and other MAPK) activations. MAPKs serve as signal integrators in most tissues (Watson et al. 2010), and examples of the details of this kind of regulation will probably be tissue specific. We have shown in the past that these signal cascades can travel down their pathways at different speeds, arriving at the final node (activated ERK) at different times (Bulayeva and Watson 2004). Summation of an early arrival at this end point with a later one, with no signals arriving in between, could generate a summed response that oscillates. Similarly, low- versus high-dose responses triggering different signaling pathways could also sum to an overall nonmonotonic curve. It is also likely that phosphatases may be specifically activated by estrogens, although this mechanism is still largely undefined (Fujita et al. 2010; Zivadinovic and Watson 2005), or other receptors might contribute. Oscillating temporal patterns can encode important information for some hormonal responses (e.g., growth hormone, gonadotropins). A role for information-rich oscillating patterns of different frequency and amplitude has recently been suggested for phosphorylations of signaling kinases and their targets (Fujita et al. 2010).

Exposed human and animal populations will probably have windows of vulnerability based on sex, life stage, and tissue differences in the expression and activities of the ERs involved in these actions. We have examined only limited cases, where ERα and perhaps GPER are involved in causing some kinase activations/deactivations. However, many other estrogen-induced signaling cascades (Belcheva and Coscia 2002; Watson and Gametchu 2003) need to be investigated in similar detail to be able to start generalizing about the mechanisms of endocrine disruption caused by XEs of particular structural classes.

## Correction

In Figures 5 and 6 in the original manuscript published online, the figures were correct but the wrong cell treatments were listed in the figure legends. The figure legends have been corrected here.
